# Influence of Frailty on Discharge Setting for Older Adults With Hip Fracture at Inpatient Rehabilitation Facilities

**DOI:** 10.1093/geroni/igaa057.570

**Published:** 2020-12-16

**Authors:** Christine Tocchi, Shamatree Shakya, Sathya Amarasekara, Michael Cary

**Affiliations:** Duke University, Durham, North Carolina, United States

## Abstract

Inpatient rehabilitation Facilities (IRFs) provide intensive rehabilitation therapy to patients to reduce functional impairment, enhance independence and return patients back to the community. Determination of eligibility for IRF is currently based on a preadmission screening. Frailty, a pervasive characteristic in older adults with hip fractures has not been examined as a clinical factor influencing function and discharge destination IRF outcomes. This study purpose was to determine the prevalence of frailty among older adult IRF patients with hip fractures and determine the association between frailty and function and discharge destination among IRF hip fracture patients. A retrospective cohort study design using CMS 2014 Inpatient Rehabilitation Facility-Patient Assessment Instrument file. Frailty was measured using a Frailty Index of 30 items. The final sample included 26,134 patients. Frailty, pre-frailty, and nonfrailty were present in 0.92% (n=24043), 3.3% (n=862), and .076% (n=199) of hip fracture patients, respectively. The majority (65%) of the patients were discharged home. There were significantly greater proportion of females than males discharged home and those of white race, 65 to 74 years of age, and those with higher functional status. Regression analysis showed significantly lower functional status at discharge (p < .0001) for males and those of non-white race, older age and frail. Study implications include the use of frailty status to identify hip fracture patients at high risk for adverse outcomes and need for future studies to explore the potential of frailty to provide value-added utility to IRF clinical settings and identify ongoing opportunities to guide person-centered care.

